# The Predictive Value of the Left Atrial Kinetic Energy for Atrial Fibrillation Recurrence

**DOI:** 10.7759/cureus.28714

**Published:** 2022-09-03

**Authors:** Sencer Çamcı, Hasan Arı, Selma Arı, Mehmet Melek, Tahsin Bozat

**Affiliations:** 1 Cardiology, Bursa Postgraduate Hospital, Bursa, TUR

**Keywords:** echocardiography, left atrial kinetic energy, recurrence, cardioversion, atrial fibrillation

## Abstract

Background and aim

Determining which patients will experience recurrence of atrial fibrillation (AF) is crucial for treatment modification. This study aimed to investigate the predictive value of left atrial kinetic energy (LAKE) in AF recurrence.

Materials and methods

A total of 120 consecutive patients who achieved sinus rhythm (SR) with electrical direct current cardioversion and met the inclusion criteria were included in the study. Transthoracic echocardiography (TTE) and LAKE values were calculated on the first day after cardioversion. Rhythm control was performed with 12-lead electrocardiography in the first-month follow-up.

Results

While 81 (67.5%) patients were in SR at one month, AF recurrence was detected in 39 (32.5%) patients. In the AF group, AF duration, cardioversion energy, number of diabetic patients, left atrium (LA) diameter, LA pre-mitral A wave volume, LA minimum volume, and pulmonary artery pressure values were significantly higher than in the SR group, while mitral A wave velocity and LAKE values were significantly lower. In multivariate regression analysis, AF duration (OR: 1.54; 95% CI: 1.22 - 1.93; p < 0.001), LA diameter (OR: 1.33; 95% CI: 1.10 - 1.61; p = 0.002), and LAKE (OR: 0.96; 95% CI: 0.94 - 0.99; p = 0.007) were determined to be independent predictors of AF recurrence at one month.

Conclusions

LA diameter, AF duration, and LAKE were found to be significant predictors of AF recurrence after cardioversion.

## Introduction

Atrial fibrillation (AF) is a common rhythm disorder among the general population and is associated with stroke, decreased exercise capacity, and increased mortality [1]. Ensuring and maintaining normal sinus rhythm (SR) is critical for reducing these negative outcomes [2].

AF begins as a result of hemodynamic or structural changes in the left atrium (LA), and during this paroxysmal and persistent phase, LA dilatation occurs and mechanical functions gradually deteriorate [3,4]. An improved understanding of LA structure and function can help to predict the risk of developing AF and response to treatment [5]. Electrical direct current cardioversion is a frequently used method of restoring SR, but a significant proportion of patients later develop AF recurrence [6]. Determining which of these patients is prone to recurrence is important for treatment modification. For this reason, the ability to predict recurrence with simple and objective parameters is valuable to clinicians. In recent years, numerous studies have been conducted on the noninvasive evaluation of LA size and mechanical function. LA function can be assessed using two-dimensional echocardiography, Doppler analysis of transmitral flow, and tissue Doppler assessment of LA myocardial velocities [7].

One of the most important factors for SR continuity is good atrial mechanical function. Left atrial kinetic energy (LAKE) is a parameter that shows left atrial mechanical function. LA size shows the anatomical specialty of LA; however, LAKE shows a combination of LA anatomical and functional specialties. LAKE can be calculated noninvasively by transthoracic echocardiography (TTE) [8]. In this study, we aimed to investigate the effectiveness of LAKE in predicting recurrence in the first month of follow-up in AF patients converted to SR with electrical direct current cardioversion.

This article was previously posted to the Research Square preprint server on April 1, 2022.

## Materials and methods

Study population

A total of 120 consecutive patients aged 18 years and older who underwent successful electrical cardioversion following persistent AF were included in the study. Persistent AF was defined as continuous AF lasting longer than seven days in electrocardiography (ECG) follow-up [9]. Patients with significant valvular disease, previous valve surgery, severe left ventricular systolic dysfunction (ejection fraction < 40%), severely dilated LA (>5 cm), previous ablation of AF, or paroxysmal AF were excluded from the study. This study followed the principles stated in the Declaration of Helsinki and was approved by the ethics committee of Bursa Postgraduate Hospital. Written informed consent was taken from all participants.

The physical examination results, medications, and laboratory results of all patients were recorded. Patients with systolic blood pressure ≥ 140 mmHg and/or diastolic blood pressure ≥ 90 mmHg or using antihypertensive medication were defined as hypertensive; patients with two consecutive fasting blood glucose measurements ≥ 126 mg/dl or using oral antidiabetic/insulin were defined as diabetic. Cerebrovascular accident (CVA), chronic renal failure (CRF), chronic obstructive pulmonary disease (COPD), and peripheral arterial disease (PAD) were recorded. ECG data (Cardiofax M ECG-1350K, Nihon Kohden, Tokyo, Japan) of the patients were recorded before and after cardioversion and one month later. The patients were divided into two groups: patients who remained in SR (group 1) and patients with AF recurrence (group 2) after one month of follow-up.

Echocardiography

All patients underwent routine TTE before cardioversion and transesophageal echocardiography (TEE) (EPIQ 7 Echocardiography Machine, Philips Ultrasound, Amsterdam, Netherlands) to exclude left atrial and left atrial appendage thrombus. TTE was repeated using a 3.5-MHz probe in patients who remained in SR 24 hours after cardioversion. All standard measurements were taken from the parasternal long and short axes and apical two- and four-chamber windows. All assessments and measurements were made according to the American Society of Echocardiography (ASE) and the European Association of Cardiovascular Imaging guidelines [10].

Left ventricular ejection fraction (LVEF) was calculated according to the modified biplane Simpson method [10]. Mitral flow velocities were recorded from the apical four-chamber view with a sample volume of 5 mm placed at the level of the mitral valve tips using pulsed wave Doppler (PWD). Peak early (E) and late (A) mitral entry velocities were recorded.

**Figure 1:**
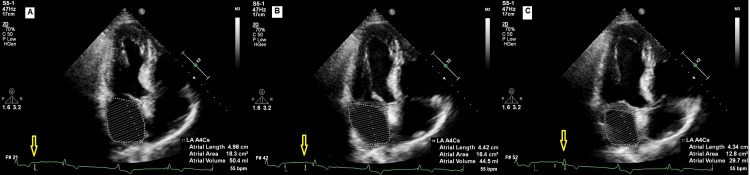
(A) Maximal LA volume just before the opening of the mitral valve at end-systole (ECG: at the end of the T wave). (B) Pre-A LA volume just before atrial systole (ECG: at the beginning of P wave). (C) Minimal LA volume at end-diastole when the mitral valve is closed (ECG: at the beginning of QRS) LA, left atrium.

LAKE values were calculated at the 24th hour after cardioversion. LAKE was defined as 0.5 × LA stroke volume (cm³, volume at the beginning of left atrial systole - LA minimal volume) × 1.06 (g/cm³, blood density) × (peak A velocity)² (cm/sec, transmitral PWD A velocity) [8]. LA volumes were measured by the biplane area-length method from the apical four-chamber view. Measured LA volumes were maximal LA volume just before the opening of the mitral valve at end-systole (ECG: at the end of the T wave), the pre-A LA volume just before atrial systole (ECG: at the beginning of P wave), and the minimal LA volume at end-diastole when the mitral valve is closed (ECG: at the beginning of QRS) (Figure 1).

LA ejection fraction (%) was calculated according to the following formula: LA maximum volume (ml) - LA minimum volume (ml)/LA maximum volume (ml).

Cardioversion

Precardioversion anticoagulation with continuous intravenous (IV) heparin infusion was applied to the patients with an activated partial thromboplastin time (aPTT) of 1.5-2 times the normal value. The patients were already anticoagulated, heparin infusion was applied to patients with international normalized ratio (INR) values ​​below 2, and cardioversion was performed when the target aPTT value (1.5-2 times the normal value) was reached. Heparin infusion was given before the procedure in patients receiving direct oral anticoagulation, and cardioversion was performed when the target (aPTT value: 1.5-2 times the normal value) was reached. Heparin infusion was stopped after cardioversion and continued with direct oral anticoagulation treatment. Patients without intracardiac thrombus on TTE and TEE were started on amiodarone infusion (15 mg/minute IV loading dose infused in 10 minutes, followed by 0.5 mg/minute infusion over 24 hours) and IV sedation with midazolam was used. The initial transthoracic electrical cardioversion energy level was set at 150 J and subsequent levels were 200 J and 270 J. External biphasic cardioversion shocks were applied at the physician’s discretion until the highest energy level was reached (270 J) or until SR was restored, in the coronary intensive care unit. Cardioversion was considered successful if the atrial P-waves persisted five minutes after shock. Oral anticoagulants were given to patients with SR for at least four weeks after the procedure [9]. The patients received antiarrhythmic treatment with oral amiodarone for a total of four weeks (600 mg/day for two weeks and 200 mg/day for the following two weeks). All patients received amiodarone to standardize the antiarrhythmic treatment. The patients were evaluated with physical examination and ECG measurements one month after the procedure; however, they were advised to come immediately if they had palpitations or symptoms of arrhythmia. If the patients had palpitation symptoms and the ECG records were SR, the patients were evaluated for AF with rhythm Holter monitoring.

Statistical analysis

Statistical analysis was performed using the Statistical Package for the Social Sciences (SPSS) computer program (version 22, IBM Corp., Armonk, NY). Continuous variables were reported as mean ± standard deviation and categorical variables as percentages. The Student's t-test was used to compare the normally distributed variables, and the Mann-Whitney U-test was used to compare the non-normally distributed variables. Categorical variables were compared with the chi-square test or Fisher's exact test as appropriate. Univariate and multivariate logistic regression analyses were used to identify significant predictors of AF recurrence following cardioversion. We performed two multivariate models for avoiding overfitting: first, include the parameters in the multivariate analysis if the p-value is smaller than 0.05; second, include the parameters in the multivariate analysis if the p-value is smaller than 0.01. A value of p < 0.05 was considered significant.

## Results

In the study, which included 120 patients who underwent successful cardioversion, 81 patients (67.5%) remained in SR at the end of the first month, while AF recurrence occurred in 39 (32.5%) patients. The duration of AF was found to be longer in group 2 than in group 1 (6.92 ± 3.15 vs. 4.74 ± 1.96 months; p < 0.001). Cardioversion energy was higher in the AF group (244.87 ± 34.01 vs. 226.66 ± 37.34 joules; p = 0.01). Diabetic patients were found more frequently in group 1 (19, 23.5%) than in group 2 (2, 5.1%) (p = 0.01). The other baseline parameters were found similar between the two groups (Table 1).

**Table 1:** Baseline characteristics of the patients SR, sinus rhythm; AF, atrial fibrillation; COPD, chronic obstructive pulmonary disease; ACEI, angiotensin-converting enzyme inhibitor; ARB, angiotensin receptor blocker.

	Group 1 (SR), n = 81	Group 2 (AF), n = 39	P-value
Age	62.16 ± 7.39	63.07 ± 7.47	0.52
Gender, n (%) (male/female)	26(% 32,1) / 55 (%67,9)	11(%28,2) / 28(%71,8)	0.66
AF duration (months)	4.74 ± 1.96	6.92 ± 3.15	<0.001
Heart rate (bpm)	109.46 ± 23.91	105.02 ± 25.27	0.35
Body mass index (kg/m²)	29.12 ± 5.61	28.89 ± 5.02	0.83
Blood pressure (mmHg)			
Systolic	129.97 ± 18.04	132.25 ± 13.29	0.48
Diastolic	81.92 ± 13.58	80.71 ± 8.40	0.61
Hemoglobin (g/dl)	13.46 ± 1.40	13.47 ± 1.50	0.97
Urea (mg/ml)	34.46 ± 10.31	35.51 ± 7.69	0.57
Creatinine (mg/ml)	0.80 ± 0.26	0.80 ± 0.24	0.96
Cardioversion energy (J)	226.66 ± 37.34	244.87 ± 34.01	0.01
Comorbidities			
Diabetes mellitus	19 (23.5%)	2 (5.1%)	0.01
Hypertension	56 (69.1%)	25 (64.1%)	0.58
COPD	5 (6.2%)	4 (10.3%)	0.42
Coronary artery disease	10 (12.3%)	5 (12.8%)	0.94
Smoker	7 (8.6%)	3 (7.7%)	0.86
Medications			
Acetylsalicylic acid	55 (67.9%)	27 (69.2%)	0.88
Beta-blocker	41 (50.6%)	25 (64.1%)	0.16
Calcium channel blocker	19 (23.5%)	8 (20.5%)	0.71
ACEI	26 (32.1%)	14 (35.9%)	0.67
ARB	22 (27.2%)	9 (23.1%)	0.63
Diuretic	3 (3.7%)	6 (15.4%)	0.06

In Table 2, the pre-cardioversion values of the groups and the control echocardiographic measurements at the 24th hour after cardioversion are compared. LA diameter (44.99 ± 2.46 vs. 42.90 ± 3.58 mm; p = 0.02), LA minimal volume (31.46 ± 13.84 vs. 23.90 ± 8.06 ml; p = 0.003), and pulmonary artery pressure (PAP) (39.64 ± 9.89 vs. 32.95 ± 8.54 mmHg; p = 0.001) were higher in AF compared to SR group; control mitral A velocity (0.62 ± 0.18 vs. 0.50 ± 0.13 m/s; p = 0.001), control left ventricle (LV) lateral Aa velocity (0.07 ± 0.02 vs. 0.05 ± 0.02 m/s; p = 0.01), and LA kinetic energy (5.36 ± 3.80 vs. 3.65 ± 2.04 kdynes.cm; p = 0.002) were higher in the SR group.

**Table 2:** Comparison of echocardiographic measurements of the groups before and after cardioversion SR, sinus rhythm; AF, atrial fibrillation; LV, left ventricle; LA, left atrium.

	Group 1 (SR), n = 81	Group 2 (AF), n = 39	P-value
LV end-systolic diameter (mm)	30.56 ± 3.65	31.33 ± 4.28	0.31
LV end-diastolic diameter (mm)	48.08 ± 3.12	48.12 ± 2.80	0.95
Septal wall thickness (mm)	11.25 ± 1.22	11.30 ± 1.25	0.84
Posterior wall thickness (mm)	10.92 ± 0.84	10.97 ± 0.93	0.77
LV ejection fraction (%)	60.95 ± 6.16	59.17 ± 6.19	0.14
LA diameter (mm)	42.90 ± 3.58	44.99 ± 2.46	0.002
LA maximum volume (ml)	84.83 ± 25.25	90.73 ± 22.31	0.21
LA volume index (ml/m^2^)	45.72 ± 13.87	49.67 ± 13.22	0.13
LA pre-A volume (ml)	33.29 ± 11.37	38.60 ± 12.73	0.02
LA minimum volume (ml)	23.90 ± 8.06	31.46 ± 13.84	0.003
LA ejection fraction (%)	0.58 ± 0.14	0,55 ± 0.15	0.24
Pulmonary artery pressure (mmHg)	32.95 ± 8.54	39.64 ± 9.89	0.001
Mitral E velocity (m/s)	1.02 ± 0.25	1.12 ± 0.28	0.06
LV lateral Ea velocity (m/s)	0.12 ± 0.03	0.13 ± 0.03	0.25
LV lateral E/Ea ratio	9.10 ± 4.33	9.18 ± 3.34	0.92
LV lateral S velocity (m/s)	0.07 ± 0.02	0.06 ± 0.02	0.35
Control mitral A velocity (m/s)	0.62 ± 0.18	0.50 ± 0.13	0.001
Control LV lateral Ea velocity (m/s), control LV lateral S velocity (m/s)	0.12 ± 0.03, 0.07 ± 0.03	0.11 ± 0.03, 0.08 ± 0.05	0.51, 0.23
Control LV lateral Aa velocity (m/s)	0.07 ± 0.02	0.05 ± 0.02	0.01
LA kinetic energy (kdynes.cm)	5.36 ± 3.80	3.65 ± 2.04	0.002

In a univariate regression analysis, diabetes mellitus (OR: 5.66; 95% CI: 1.24 - 25.73; p = 0.02), AF duration (OR: 1.39; 95% CI: 1.18 - 1.63; p < 0.001), cardioversion energy (OR: 1.01; 95% CI: 1.003 - 1.02; p = 0.01), LA diameter (OR: 1.22; 95% CI: 1.07 - 1.39; p = 0.02), LV lateral Aa velocity (OR: 0.001; 95% CI: 0.0001 - 0.017; p = 0.01), PAP (OR: 1.08; 95% CI: 1.03 - 1.12; p = 0.001), and LAKE (OR: 0.98; 95% CI: 0.96 - 0.99; p = 0.001) were found to be significant predictors of AF recurrence. In a multivariate regression analysis, AF duration (OR: 1.63; 95% CI: 1.25 - 2.13; p < 0.001), LA diameter (OR: 1.37; 95% CI: 1.10 - 1.70; p = 0.004), and LAKE (OR: 0.74; 95% CI: 0.56 - 0.97; p = 0.03) were found to be independent predictors of AF recurrence at one month (Table 3). In the second multivariate logistic regression model, again the AF duration (OR: 1.54; 95% CI: 1.22 - 1.93; p < 0.001), LA diameter (OR: 1.33; 95% CI: 1.10 - 1.61; p = 0.002), and LAKE (OR: 0.96; 95% CI: 0.94 - 0.99; p = 0.007) were independent predictors of AF recurrence at one month (Table 3).

**Table 3:** Evaluation of parameters with univariate and multivariate logistic regression analysis in terms of predicting AF recurrence in the first month OR, odds ratio; CI, confidence interval; AF, atrial fibrillation; LA, left atrium; LV, left ventricle; LVEF, left ventricular ejection fraction; PAP, pulmonary artery pressure; LAKE, left atrial kinetic energy.

Variable	Univariate logistic regression analysis		Multivariate logistic regression analysis			
			Model 1		Model 2	
	OR (95% CI)	P-value	OR (95% CI)	p-Value	OR (95% CI)	P-value
Age (year)	1.01 (0.96 - 1.07)	0.52				
Gender	0.83 (0.35 - 1.92)	0.66				
Diabetes mellitus	5.66 (1.24 - 25.73)	0.02	7.48 (0.96 - 8.02)	0.054		
AF duration (months)	1.39 (1.18 - 1.63)	<0.001	1.63 (1.25 - 2.13)	<0.001	1.54 (1.22 - 1.93)	<0.001
Cardioversion energy (J)	1.01 (1.003 - 1.02)	0.01	1.007 (0.99 - 1.02)	0.31		
LA diameter (cm)	1.22 (1.07 - 1.39)	0,002	1.37 (1.10 - 1.70)	0.004	1.33 (1.10 - 1.61)	0.002
LA ejection fraction (%)	0.20 (0.015 - 2.94)	0.24				
LV lateral Aa velocity (m/s)	0.001 (0.0001 - 0.017)	0.01	0.001 (0.0001 - 9.09)	0.12		
LVEF (%)	0.95 (0.89 - 1.01)	0.16				
PAP (mmHg)	1.08 (1.03 - 1.12)	0.001	1.03 (0.97 - 1.10)	0.28	1.05 (0.99 - 1.12)	0.06
LAKE	0.98 (0.96 - 0.99)	0,001	0.74 (0.56 - 0.97)	0.03	0.96 (0.94 - 0.99)	0.007

## Discussion

In this study, we investigated the value of LAKE, an indicator of left atrial mechanical function, in predicting AF recurrence one month later in patients with persistent AF who underwent SR by applying electrical direct current cardioversion. LAKE was found to be significantly lower in the AF group than in the SR group, and LAKE, LA diameter, and AF duration were found to be independent predictors of AF recurrence at one month.

LAKE is an important indicator of the contribution of the LA in the left ventricular filling. In patients with abnormal diastolic dysfunctions, such as hypertrophic cardiomyopathy, the ventricular filling is impaired. As a compensatory mechanism to overcome this situation, left atrial contractility may increase as a result of the Frank-Starling mechanism, which provides adequate left ventricular filling [11]. Similarly, in patients with mitral stenosis, there is a compensatory increase in LA mechanical function against the stenotic valve before left atrial insufficiency develops [12]. However, when mitral stenosis progresses and becomes symptomatic, LAKE gradually decreases instead of increasing due to persistent loading in the LA [13]. Again, in patients with heart failure, especially in patients with mild to moderate heart failure symptoms, an increase in LA mechanical function occurs to compensate for the decreased left ventricular function [14]. However, as heart failure progresses, left atrial function also deteriorates, and LAKE decreases [13,15]. In a study performed by Chrysohoou et al. [16], six-month follow-ups revealed a higher frequency of cardiovascular events in patients with newly diagnosed left ventricular failure and impaired LA mechanical function.

In a study comparing two groups consisting of paroxysmal AF patients and normal individuals, no significant difference was found between the two groups in terms of LAKE [17]. One of the issues discussed in the study was the exclusion of persistent/permanent AF patients. This is an issue because deterioration in LA mechanical function is expected to be more common in the chronic stage.

As discussed above, LA is an attempt to compensate for the stroke volume by increasing its mechanical function as a result of Frank-Starling mechanisms to overcome the problems in the left ventricle or valve [11,12]. However, after a while, especially as the diseases progress, the LA structure begins to deteriorate, and mechanical function decreases [13]. Deteriorated LA is more prone to the development of AF. LAKE, which is an important indicator of LA mechanical function, also decreases over time. In our study, LAKE was found to be lower in patients with AF recurrence at one-month follow-up after cardioversion.

LA mechanical function is not restored immediately after cardioversion, this phenomenon is named "atrial stunning" [18]. LA gains its mechanical activity within the first 24 hours, in 80% of patients [19]. Kinetic energy has been shown to provide insight into LA contractile function. The early recovery of LA functions reduces the recurrence of AF. The duration of AF, LA dimension, and the percent increase of the transmitral A-wave velocity from four to 24 hours have predictive value for the long-term success of cardioversion [20]. In our study, we showed that a lower LAKE value had a predictive value for AF recurrence in one month. The prediction value is independent of the other AF recurrence predictor parameters.

LA diameter is one of the parameters that predict AF recurrence at one month in multivariate analysis. Decreased atrial function and AF recurrence are expected to occur in patients with an enlarged LA diameter, and this has also been shown in previous studies [3,4]. Kuppahally et al. showed that the LA diameter was 44.7 mm in the persistent AF group and 37.6 mm in the paroxismal AF group [4]. In our study, we showed that the LA diameter was 44.9 mm in the AF recurrence group and 42.9 mm in the SR group. The anteroposterior diameter of the LA was measured in our patients, but the three-dimensional anatomy of the LA may provide more valuable information. The fact that LAKE is a predictor of AF recurrence independent of LA diameter is an indicator that LA function cannot be evaluated with LA diameter alone.

AF duration is another independent predictor of AF recurrence. As the duration of AF increases, LV mechanical function gradually worsens, atrial fibrosis increases, and AF recurrence increases in patients undergoing cardioversion. Especially in patients with an AF duration of three months or more, atrial functions begin to decrease, and AF recurrence increases [21]. In our study, the AF duration was found to be longer in the group with AF recurrence. In addition, the high rate of AF recurrence (32.5%) seen at one month can be explained by the long AF durations in our study group. The duration of AF was 4.74 months in the SR group and 6.92 months in the AF group. A previous study showed that greater than three months of AF duration was an independent predictor for AF recurrence; in our study, the AF duration was 6.92 months in the AF recurrence group [21]. The AF recurrence rate was similar to our study, as one-third of the study population had AF recurrence.

Clinical implications

It has been shown that LAKE is reduced in patients with AF recurrence. Due to the success of LAKE in showing one-month short-term recurrences, AF recurrence may be prevented by applying more aggressive treatment in patients with low LAKE values.

Limitations

The relatively small number of patients is the first limitation of our study. Due to this limited number of patients, the study may not be representative of the entire population. In addition, the follow-up period is as short as one month. It should be investigated whether the one-month follow-up results are an indicator of the success of LAKE in predicting AF recurrence in long-term follow-ups. The follow-up was performed with physical examination and ECG; however, rhythm Holter monitoring may show us the paroxysmal AF patients.

## Conclusions

LAKE, AF duration, and LA diameter were found to be significant predictors of AF recurrence one month after cardioversion. LAKE, an important indicator of left atrial mechanical function, predicts AF recurrence one month after successful electrical cardioversion in patients with persistent AF. In patients with low LAKE values, more aggressive treatment may be considered for the maintenance of SR. The results of this study, in which LAKE was identified as an independent predictor of AF recurrence, should be confirmed by studies with larger patient groups and longer follow-up periods.

## References

[1] Stewart S, Hart CL, Hole DJ, McMurray JJ (2002). A population-based study of the long-term risks associated with atrial fibrillation: 20-year follow-up of the Renfrew/Paisley study. Am J Med.

[2] Chung MK (2004). Randomized trials of rate vs. rhythm control for atrial fibrillation. J Interv Card Electrophysiol.

[3] Lim DJ, Ambale-Ventakesh B, Ostovaneh MR (2019). Change in left atrial function predicts incident atrial fibrillation: the Multi-Ethnic Study of Atherosclerosis. Eur Heart J Cardiovasc Imaging.

[4] Kuppahally SS, Akoum N, Burgon NS (2010). Left atrial strain and strain rate in patients with paroxysmal and persistent atrial fibrillation: relationship to left atrial structural remodeling detected by delayed-enhancement MRI. Circ Cardiovasc Imaging.

[5] Sitges M, Teijeira VA, Scalise A (2007). Is there an anatomical substrate for idiopathic paroxysmal atrial fibrillation? A case-control echocardiographic study. Europace.

[6] Ari H, Ari S, Sarigül OY (2016). A novel index combining diastolic and systolic tissue Doppler parameters for prediction of atrial fibrillation recurrence. Echocardiography.

[7] Kurt M, Wang J, Torre-Amione G, Nagueh SF (2009). Left atrial function in diastolic heart failure. Circ Cardiovasc Imaging.

[8] Boudoulas H, Boudoulas D, Sparks EA, Pearson AC, Nagaraja HN, Wooley CF (1995). Left atrial performance indices in chronic mitral valve disease. J Heart Valve Dis.

[9] Hindricks G, Potpara T, Dagres N (2021). 2020 ESC guidelines for the diagnosis and management of atrial fibrillation developed in collaboration with the European Association for Cardio-Thoracic Surgery (EACTS): the Task Force for the Diagnosis and Management of Atrial Fibrillation of the European Society of Cardiology (ESC) developed with the special contribution of the European Heart Rhythm Association (EHRA) of the ESC. Eur Heart J.

[10] Lang RM, Badano LP, Mor-Avi V (2015). Recommendations for cardiac chamber quantification by echocardiography in adults: an update from the American Society of Echocardiography and the European Association of Cardiovascular Imaging. Eur Heart J Cardiovasc Imaging.

[11] Anwar AM, Soliman OI, Nemes A, Geleijnse ML, ten Cate FJ (2008). An integrated approach to determine left atrial volume, mass and function in hypertrophic cardiomyopathy by two-dimensional echocardiography. Int J Cardiovasc Imaging.

[12] Boudoulas KD, Sparks EA, Rittgers SE, Wooley CF, Boudoulas H (2003). Factors determining left atrial kinetic energy in patients with chronic mitral valve disease. Herz.

[13] Stefanadis C, Dernellis J, Lambrou S, Toutouzas P (1998). Left atrial energy in normal subjects, in patients with symptomatic mitral stenosis, and in patients with advanced heart failure. Am J Cardiol.

[14] Triposkiadis F, Harbas C, Sitafidis G, Skoularigis J, Demopoulos V, Kelepeshis G (2008). Echocardiographic assessment of left atrial ejection force and kinetic energy in chronic heart failure. Int J Cardiovasc Imaging.

[15] Dernellis JM, Panaretou MP (2003). Effects of digoxin on left atrial function in heart failure. Heart.

[16] Chrysohoou C, Kotroyiannis I, Antoniou CC (2014). Left atrial function predicts heart failure events in patients with newly diagnosed left ventricular systolic heart failure during short-term follow-up. Angiology.

[17] Yoon YE, Kim HJ, Kim SA (2012). Left atrial mechanical function and stiffness in patients with paroxysmal atrial fibrillation. J Cardiovasc Ultrasound.

[18] Logan WF, Rowlands DJ, Howitt G, Holmes AM (1965). Left atrial activity following cardioversion. Lancet.

[19] Silverman DI, Manning WJ (1998). Role of echocardiography in patients undergoing elective cardioversion of atrial fibrillation. Circulation.

[20] Dethy M, Chassat C, Roy D, Mercier LA (1988). Doppler echocardiographic predictors of recurrence of atrial fibrillation after cardioversion. Am J Cardiol.

[21] Fornengo C, Antolini M, Frea S, Gallo C, Grosso Marra W, Morello M, Gaita F (2015). Prediction of atrial fibrillation recurrence after cardioversion in patients with left-atrial dilation. Eur Heart J Cardiovasc Imaging.

